# Through Thick and Thin: Identifying Barriers to Bariatric Surgery, Weight Loss Maintenance, and Tailoring Obesity Treatment for the Future

**DOI:** 10.1155/2016/8616581

**Published:** 2016-05-23

**Authors:** Donevan Westerveld, Dennis Yang

**Affiliations:** ^1^Department of Internal Medicine, University of Florida College of Medicine, Gainesville, FL 32601, USA; ^2^Division of Gastroenterology, University of Florida College of Medicine, Gainesville, FL 32601, USA

## Abstract

More than one-third of the adults in the United States are obese. This complex metabolic disorder is associated with multiple comorbidities and increased all-cause mortality. Bariatric surgery has been shown to be more effective than medical therapy and has been associated with weight loss maintenance and decreased mortality. In spite of these well-established benefits, less than 1% of candidates undergo surgery due to multiple factors, such as patient and physician perceptions and attitudes, patient-physician interaction, lack of resources, and cost burden. Furthermore, even in patients who do undergo bariatric surgery and/or alternate weight loss interventions, long-term weight control is associated with high-risk failure and weight regain. In this review, we highlight some of the current barriers to bariatric surgery and long-term weight loss maintenance and underscore the importance of an individualized multidisciplinary longitudinal strategy for the treatment of obesity.

## 1. Introduction

The pandemic of our generation is undoubtedly the rise and prevalence of obesity. Some of the strongest statistical evidence concerning obesity rates comes from the National Health and Nutrition Examination Survey (NHANES) with their most recent report indicating that an estimated 33.9% of US adults aged 20 or older are overweight (BMI 25.0–29.9 kg/m^2^), 35.1% are considered obese (BMI ≥ 30 kg/m^2^), and 6.4% are considered morbidly obese (BMI ≥ 35 kg/m^2^) [1, 2]. From a global perspective, an estimated 1.48 billion adults are thought to be overweight of which 502 million individuals are classified as obese [3, 4].

Obesity is a complex metabolic disorder associated with multiple comorbidities, most notably type 2 diabetes mellitus, all cardiovascular diseases, hypertension, nonalcoholic fatty liver disease, obstructive sleep apnea, certain malignancies, and increased all-cause mortality [5–7]. In aggregate, this has an enormous impact on quality of life and imposes a significant threat to the economy of our health care system. Indeed, obesity and the aforementioned comorbidities have accounted for an estimated 27% growth in US health care expenditure [8]. Moreover, it is estimated that 16%–18% ($66 billion/year) of total US healthcare expenditure will be used to treat those overweight and obese [9].

Current accepted therapies for obesity and associated metabolic comorbidities include lifestyle modification (i.e., behavioral changes, diet, and physical activity), pharmacological agents, and surgical intervention. Lifestyle modification as a standalone therapy has limited effectiveness with 5% to 10% total body weight loss at 1 year and high rates of weight recidivism [10–12]. Indeed, studies have demonstrated that most patients regain 33%–50% of their weight within the first year and regain almost all of their weight by the second year [13–15]. Moreover, the usage of pharmacological agents, such as orlistat, diethylpropion, and phendimetrazine, has been limited by low rates of compliance and adverse effects [16, 17].

Bariatric surgery remains the most effective and durable therapy option for obesity. Bariatric surgery is generally considered when nonsurgical interventions have failed in a patient with a BMI of ≥35 kg/m^2^ with one or more comorbidities, or a BMI of ≥40 kg/m^2^ [18, 19]. Common bariatric surgeries include Roux-en-Y gastric bypass (RYGB), sleeve gastrectomy, and adjustable gastric band. A recent meta-analysis demonstrated an overall percent excess weight loss (%EWL) (amount of weight loss/(patient's initial weight − ideal body weight) × 100) of 59.8% (range 50.5%–69.2%) following bariatric surgery [20]. Bariatric surgery not only is associated with weight loss maintenance but also improves obesity-related comorbidities and decreases mortality [19, 21]. In a recent study with a 10-year follow-up period, patients who underwent bariatric surgery had a significantly greater improvement in their comorbidities when compared to patients who did not have surgery [22]. The effects of bariatric surgery are not exclusively limited to weight loss and the improvement of comorbidities but have far-reaching ramifications on our health care system as well. The long-term cost-effectiveness of bariatric surgery has been previously estimated in various studies [23]. If we take into account only the cost of treating type 2 diabetes mellitus in the obese population, we could expect direct 10-year aggregate cost savings of $2.7 million/1000 people. The indirect cost, which takes into account the cost paid by society in terms of loss of work productivity due to sick leave or disability, has been proposed at $5.4 million/1000 [24]. Moreover, others have estimated the indirect cost of obesity to cost our society $48 billion to $64 billion annually [25, 26].

In spite of the proven benefits of bariatric therapy, surgery remains a scarcely pursued intervention for those individuals who qualify for the procedure [27]. Indeed, it is estimated that less than 1% of candidates undergo surgical intervention [28–30]. There are multiple factors that may contribute to the underutilization of bariatric surgical services. Furthermore, even in patients who do undergo bariatric surgery and/or alternate weight loss interventions, long-term weight control is associated with high-risk failure and weight regain. In this review, we highlight some of the current barriers to bariatric surgery and long-term weight loss maintenance and underscore the importance of an individualized multidisciplinary longitudinal strategy for the treatment of obesity.

## 2. Barriers to Bariatric Surgery

### 2.1. Patient Demographics

There are several factors associated with the underutilization and high attrition rate in most bariatric surgical programs. Patient characteristics play a significant role and should be recognized in order to implement effective countermeasures. In a recent retrospective study of 1682 patients undergoing a publicly funded bariatric surgery program, both male sex and age of 60 years or older were variables associated with recidivism [31]. Male patients appear to be less receptive to bariatric surgery in general as other studies have demonstrated women to have a more positive outlook on the safety and risks associated with bariatric surgery when compared to their male peers [31, 32]. In addition to gender and age, psychological characteristics have also been associated with bariatric surgery outcomes. As such, presurgical psychiatric assessment is a vital piece in the standard evaluation of any patient prior to bariatric surgery [33, 34]. Previous studies have identified that one-fifth of patients undergoing presurgical evaluation are not eligible candidates due to emotional distress and a tendency to overeat as a coping mechanism [34]. Other reasons for exclusion included current uncontrolled psychopathology, current life stressors, and insufficient effort at a formal diet program. Individuals with active substance use have also been identified as less likely to successfully adhere to a bariatric surgery program [31, 34, 35]. Providers must recognize these patient factors as potential barriers before committing an individual to a bariatric surgical program. It is important to first understand the patients' attitudes and perceptions towards their disease and therapy in order to identify any misconceptions and gaps of knowledge. Once this has been addressed, the different treatment options, including bariatric surgery, should be presented to the patient. An effort should be placed on highlighting the impact of obesity on their overall health, as studies have shown that patients with higher comorbidities are more receptive to bariatric surgery [36]. Most importantly, given how patient characteristics can significantly impact their treatment outcomes, we must realize that a “single” treatment approach will be ineffective and that an individualized targeted plan needs to be formulated for each patient. This plan may include variable interventions, including structured nutritional education as well as psychiatric referral to assist with emotional stressors and substance use.

### 2.2. Physician Factors

Establishing a strong patient-physician relationship is a key cornerstone not only in the management of the obese patient but also in medicine in general. Patient information and education about their illness and treatment options are essential and should precede any medical decision-making. It is only then that an open mutual discussion can follow around the benefits, risks, costs, and alternative therapy options.

Arterburn and colleagues recently evaluated practices of severely obese patients who are not seeking bariatric surgery. In this survey study, 49% of the 295 severely obese participants indicated that they were interested in learning more about bariatric surgery and pharmacotherapy; yet, only 26% of them discussed the options with their physician [37]. While the authors did not elaborate on the reasons for the low interest and even lower active pursuit of treatment in their study population, other studies have alluded to patients' inaccurate perceptions of bariatric surgery as a cause for underutilization. In fact, in a study of 130 obese individuals with type 2 diabetes mellitus, only 20% had a favorable impression of bariatric surgery [32]. Furthermore, less than 15% and 29% of the participants perceived bariatric surgery to be safe and effective for the treatment of type 2 diabetes mellitus, respectively [32]. Overall, these studies clearly suggest that physicians need to recognize this discrepancy in utilization of bariatric therapy resources, identifying the barriers to treatment in their patient, and then jointly formulate a stepwise plan. In order for this to happen, physicians need to be informed and comfortable with discussing bariatric interventions with their obese patients.

Several studies have shown that physicians' attitudes about obesity and its treatment vary among practitioners and most of them do not directly address their obese patients' weight issues [38–40]. Indeed, most practitioners recognize the severity of this illness but have low confidence in their ability to instruct and guide their patients. In a survey study of primary care physicians' attitudes, beliefs, and referral patterns to bariatric surgery, Balduf et al. demonstrated that many nonreferring physicians felt unprepared to provide quality long-term medical care to their obese patients and less than half (45%) felt competent to address medical complications following bariatric surgery [41, 42]. These physicians were less familiar with NIH guidelines and had lower participation rates in bariatric continuing education when compared to their referring peers. Overall, the lack of resources and knowledge to manage these postbariatric surgical patients adequately was identified as one of the factors in nonreferring physicians' attitude towards adopting a noninterventional approach. These results emphasize the importance of physicians' education on obesity and its treatment options, as well as on the long-term effects of bariatric therapy and its medical management. This daunting task should not fall on the primary care practitioners' shoulders alone, but it should be undertaken as a multidisciplinary collaborative effort among all providers, including surgeons, dieticians, endocrinologists, and psychiatrists. This multidisciplinary approach is indispensable in order to ensure the long-term efficacy of any bariatric intervention and the patient's continued adherence to a regular diet and exercise regimen to prevent recidivism.

### 2.3. Financial Factors

Lack of coverage and high costs has been traditionally labeled as a major factor associated with the underutilization of bariatric surgical services. However, financial reasons may have less of a negative impact as previously stipulated [27, 29]. In a study by Afonso and colleagues evaluating perceived barriers to bariatric surgery, only 27% of the participants (*n* = 77) indicated lack of insurance coverage as the main reason. Furthermore, a publicly funded bariatric surgery program in Canada clearly showed that a lack of funding was not a significant factor in a patient's decision to undergo surgery as the attrition rate in this study was nearly 65% [31]. Interestingly, a separate survey study also demonstrated that patients with insurance coverage were less likely to have thought about having bariatric surgery [37]. Many physicians themselves perceive cost to be prohibitive as the financial burden would be too great for their patients [42]. While it is needless to say that cost needs to be factored in the medical decision-making, it is crucial that both the providers and their patients explore all possibilities and coverage options before prematurely excluding bariatric surgery as a feasible intervention. Furthermore, consideration should be taken into the direct and indirect costs associated with the ineffective treatment of obesity and its comorbidities.

## 3. Barriers to Long-Term Weight Loss Maintenance

As previously highlighted, bariatric surgery remains the most effective and durable treatment for obesity. We have identified barriers to its utilization and highlighted the importance of a multidisciplinary approach to address these issues. More importantly, we recognize that obesity is a chronic disease and the main challenge to its long-term management does not revolve around weight loss but rather around its sustainability.

There are multiple interacting factors that contribute to the high-risk failure and weight regain following bariatric medical and/or surgical therapies. Recently, the National Institutes of Health (NIH) organized a workshop to identify the challenges that make maintaining weight loss difficult [43]. Identifying these factors and instituting potential strategies to overcome these obstacles are the ongoing challenges of long-term weight control.

Several studies have attempted to elucidate factors associated with weight loss maintenance by focusing on individuals who have been successful in maintaining long-term weight control [44–48]. The National Weight Control Registry (NWCR) evaluated long-term maintenance weight loss in 629 women and 155 men who had lost an average of 30 kg and maintained a required minimum weight loss of 13.6 kg over a 5-year follow-up period [49]. Registry participants were asked to respond to a series of questions regarding demographics, weight characteristics, and weight maintenance methods and strategies. Overall, strategies associated with adequate long-term weight control included dietary modification (i.e., limit intake of certain foods, eating regular small meals, and avoiding fast foods and restaurants), adhering to daily physical activity, and self-monitoring weight. The authors concluded that, by adopting a variety of strategies (i.e., cooking different types of healthy meals and diverse physical activities ranging from walking to weightlifting), the participants were more likely to lose and maintain weight when compared to individuals who may attempt to use only one standard set of strategies. Similarly, McGuire and colleagues studied behavioral strategies of individual with maintained long-term weight loss through a random digit dial telephone survey [50]. When compared to subjects with weight regain or controls (weight-stable subjects), weight loss maintainers adopted behavioral strategies for reduction in dietary fat intake (i.e., substituting high-fat for low-fat options) and engaged in more strenuous activities (i.e., running, weightlifting, and aerobics).

There are several inherent limitations to these cross-sectional studies, including the accuracy of self-reported data and the inability to draw cause and effect conclusions between the variables and successful weight loss maintenance. Most importantly, while these reports demonstrate that adherence to a healthy diet and regular physical activity are the cornerstones for long-term weight control, this conclusion may be to some extent self-evident and does not necessarily address why most obese patients fail to do so after initial weight loss. There are many biological and behavioral factors that interact with and influence the cognitive processes involved weight loss maintenance [51]. While it has been previously suggested that part of the phenomenon of weight regain is due to the biologic driving force behind homeostasis (set-point theory) [52], studies such as the NWCR suggest that this may be overcome through behavioral strategies. Based on these findings, there has been an emphasis on evaluating how these cognitive mechanisms may contribute to long-term weight control. The QUOVADIS (quality of life in obesity: evaluation and disease surveillance) multicenter observational study evaluated several cognitive factors associated with long-term weight loss in 1944 obese patients [53]. In general, the study identified that certain cognitive factors were associated with weight loss (i.e., increased dietary restraint and reduced disinhibition) and long-term weight loss maintenance (i.e., satisfaction with results achieved and confidence in ability to maintain weight loss). On the other hand, high weight-loss expectations, appearance-based primary motivation for weight loss, and unsatisfactory progress were all associated with higher rates of attrition. More recently, Kiesewetter et al. suggested that psychological factors, such as an individual's attachment style, may play an important role in the success of long-term interventions [54]. Overall, the authors demonstrated that weight loss at 12-month follow-up was significantly higher in patients with secure attachment than in those with insecure attachment. Patients with secure attachment also rated a more positive patient-therapist relationship.

## 4. Tailoring Long-Term Weight Management: Future Directions

Several strategies have been evaluated to promote weight loss and long-term weight loss maintenance. While studies have demonstrated that there are no set strategies for all patients, it is evident that a longitudinal multidisciplinary comprehensive model may be the most effective approach in the treatment of obesity. From the initial evaluation, establishing a strong patient-physician relationship is imperative in order to identify any potential barriers to treatment, which in turn may help tailor the most appropriate intervention for the individual.

Initial strategies should be focused on lifestyle modifications, including diet and physical activity. This strategy may be potentially enhanced by adjunct pharmacotherapy. In the XENDOS (Xenical in the prevention of diabetes in obese subjects) study, weight loss maintenance at 4-year follow-up was significantly higher in patients undergoing lifestyle changes with orlistat than placebo [55]. The results from this trial demonstrated that pharmacologic agents may assist with weight loss and also reduce the incidence of diabetes. More recently, four additional drugs (lorcaserin, naltrexone/bupropion, phentermine/topiramate, and liraglutide) have been approved by the Food and Drug Administration (FDA). It is important to recognize that there are currently no head-to-head comparison trials among these pharmacological agents and thereby selection of a particular drug should be tailored to the patient's needs, tolerability, and costs. For instance, obese patients with a propensity for binge eating may benefit from naltrexone/bupropion as a prior trial demonstrated that this drug was associated with improved control over food cravings [56]. On the contrary, lorcaserin, a serotonin 2C receptor agonist, should be avoided in patients treated with selective serotonin reuptake inhibitors (SSRIs) given the increased risk of serotonin syndrome [57]. Overall, while all of these agents have achieved the effective 5% to 10% weight loss benchmarks established by the FDA, their particular role in long-term weight loss control still remains unclear.

In addition to pharmacotherapy, longitudinal supervision and support of patients following the initial weight loss phase may be even more important in promoting long-term weight loss maintenance. Svetkey et al. demonstrated that longitudinal follow-up with monthly brief personal contact of patients improved sustained weight loss [48]. In a separate study, patients undergoing a self-weighing and regulation program with face-to-face feedback endorsed less weight gain when compared to patients who received quarterly newsletters or Internet-based feedback [57]. Similarly, patients involved in self-help group sessions on a monthly basis tend to have improved success in adhering to a long-term weight loss maintenance regimen [58]. In all, these reports reiterate the notion that longitudinal support from providers as well as other individuals undergoing weight loss maintenance is crucial in perpetuating weight control behaviors among participants.

Physical activity has been clearly shown to have a significant impact on weight loss maintenance [59]. Several studies have evaluated methods to increase motivation and adherence to routine physical activity. Lee et al. demonstrated in their study that intrinsically enjoyable physical activities, such as Latin dancing, can be used to increase physical activity duration and intensity when compared to more traditional exercise regimes [60]. This represents another example of how a tailored program can assist with adherence to lifestyle changes while also providing a venue of ongoing social support.

As mentioned earlier, the success of long-term weight loss maintenance is directly associated with many cognitive factors and behavioral aspects of each individual patient. Patients should be openly engaged by a multidisciplinary team. Traditionally, most psychologists have been largely involved in the preoperative evaluation of potential bariatric surgical candidates. However, it has become more evident that their role should be expanded to include the evaluation of all obese patients undergoing weight management, as identification of barriers to treatment and training in cognitive behavioral strategies are the basis for longitudinal success. In addition, physicians need to be familiar with weight loss and maintenance strategies in order to educate and formulate a plan for their obese patients. Individuals should be actively monitored for any adverse events associated with treatment strategies and referred to other health professionals when indicated. It is only through a comprehensive multidisciplinary team approach that we may potentially address the various obstacles associated with the treatment of obesity.

## 5. Conclusion

Nearly two-thirds of our population is considered overweight or obese and if predictions bear truth we will live in a population where there are more obese Americans than not. Shortened life expectancies, increased health care expenditures, and significant comorbidities are some of the ramifications of this complex disease. Currently, bariatric surgery remains the most effective therapeutic intervention in treating obesity and its associated comorbidities. However, it remains a grossly underutilized intervention. Multiple factors, such as patient and physician perceptions and attitudes, patient-physician interaction, lack of resources, and cost burden, are all potential barriers to bariatric surgery. Even in patients who do undergo bariatric surgery and/or other alternate weight loss interventions, long-term weight loss maintenance has been the main challenge in the treatment of obesity. This review highlights the importance of recognizing these obstacles and the need to adopt a multidisciplinary approach in the management of these complex patients. The primary referring physician cannot operate in a vacuum but needs the ongoing assistance from various specialties (psychologists, endocrinologists, dieticians, and surgeons) in order to provide adequate comprehensive longitudinal care for these patients and to maximize the chance of favorable outcomes. Future prospective studies are needed to evaluate the efficacy of a structured multidisciplinary longitudinal approach.
